# Variation of peripheral blood-based biomarkers for response of anti-PD-1 immunotherapy in non-small-cell lung cancer

**DOI:** 10.1007/s12094-024-03416-5

**Published:** 2024-03-07

**Authors:** Xiaoming Wang, Dayu Chen, Yuyan Ma, Dongping Mo, Feng Yan

**Affiliations:** Department of Clinical Laboratory, Nanjing Medical University Affiliated Cancer Hospital & Jiangsu Cancer Hospital & Jiangsu Institute of Cancer Research, Baizi Ting No.42, Nanjing, 210009 Jiangsu China

**Keywords:** Non-small-cell lung cancer, Immunotherapy, Machine learning, Hematological biomarkers

## Abstract

**Purpose:**

Immune checkpoint inhibitors (ICIs) for non-small-cell lung cancer (NSCLC) are on the rise, but unfortunately, only a small percentage of patients benefit from them in the long term. Thus, it is crucial to identify biomarkers that can forecast the efficacy of immunotherapy.

**Methods:**

We retrospectively studied 224 patients with NSCLC who underwent anti-PD-1 therapy. The role of biomarkers and clinical characteristics were assessed in a prognostic model.

**Results:**

Only 14.3% of patients had both programmed death ligand 1 (PD-L1) and tumor mutational burden (TMB) outcomes, highlighting the need to investigate more available biomarkers. Our analysis found a correlation between histological PD-L1 TPS and hematological PD-1 expression. Analysis of hematological biomarkers revealed that elevated expression of CD4/CD8 and LYM% are positively associated with effective immunotherapy, while PD-1^+^ on T cells, NLR, and MLR have a negative impact. Moreover, high level of ΔCEA%, CYFRA21-1 and LDH may suggest ineffective ICIs. We also observed that disparate immunotherapy drugs didn’t significantly impact prognosis. Lastly, by comparing squamous carcinoma and adenocarcinoma cohorts, ΔCEA%, CD3^+^PD-1^+^, CD4^+^PD-1^+^, and CD4/CD8 are more important in predicting the prognosis of adenocarcinoma patients, while age is more significant for squamous carcinoma patients.

**Conclusion:**

Our research has yielded encouraging results in identifying a correlation between immunotherapy’s response and clinical characteristics, peripheral immune cell subsets, and biochemical and immunological biomarkers. The screened hematological detection panel could be used to forecast an NSCLC patient’s response to anti-PD-1 immunotherapy with an accuracy rate of 76.3%, which could help customize suitable therapeutic decision-making.

**Supplementary Information:**

The online version contains supplementary material available at 10.1007/s12094-024-03416-5.

## Introduction

Lung cancer has the highest mortality rate of 18% resulting in approximately 1.8 million deaths annually worldwide, and over 70% of patients are diagnosed in advanced stages with only a 10% to 20% five-year survival rate [1]. Recently, immune checkpoint inhibitors (ICIs) have shown significant promise, with anti-PD-1/PD-L1 immunotherapy as a typical case, which has improved progression-free survival (PFS) and overall survival (OS) for advanced NSCLC [2–4].

Despite the advantages of ICIs, only a fraction of patients experiences a durable benefit due to individual differences and tumor microenvironments [5]. Biomarkers can help us understand the state of a patient’s immune system and how the tumor is responding to immunotherapy [6–8]. One biomarker is the PD-L1’s expression from tissue [9]. A phase 3 study of first-line pembrolizumab monotherapy with locally advanced or metastatic NSCLC found that patients with a PD-L1 tumor proportion score (TPS) of 50% or greater had the lowest hazard ratio for OS [10]. Another biomarker is TMB, which is linked to improved objective response, durable clinical benefit, and PFS in NSCLC [11]. Subsequent studies have found higher TMB is associated with improved survival in most cancers [12].

However, intricate dynamics make monitoring biomarkers for cancer cells and the immune system complex. Histological inspection is invasive and unsuitable for real-time monitoring. Hematological biomarkers, including soluble PD-L1 and blood-based TMB, offer valuable real-time insights into the tumor and host microenvironment [13–16]. Moreover, peripheral immune cells like CD8^+^ T cells, CD4^+^ T cells, regulatory T cells, T-cell receptor repertoire, and neutrophil-to-lymphocyte ratio (NLR) can impact responses of ICIs [17–19]. In addition, cytokines such as interleukin-6, interleukin-8, interferon-gamma, C-reactive protein, and lactate dehydrogenase (LDH) have been identified as either predictive or prognostic factors for immunotherapy [20–22].

In this work, we performed a retrospective analysis of 224 NSCLC patients who received anti-PD-1 immunotherapy. The 14 prognostic biomarkers were identified from blood cell counts, biochemical levels, immunoassay results, and immune cell subsets. This detection panel will help us develop more personalized treatment plans to improve ICIs outcomes.

## Materials and methods

### Study population

This investigation received approval from the Clinical Research Ethics Committee of Nanjing Medical University (2023K061). All investigations have been in accordance with the principles embodied in the declaration of Helsinki. We collected all patients with recurrent or metastatic NSCLC who were receiving anti-PD-1 immunotherapy from Jiangsu Cancer Hospital in China from March 2023 to July 2023, and all precious examinations of these individuals were gathered for review. We excluded patients who received less than three treatment cycles or those with unevaluable responses. In the course of our information collection, it can be found that less than 2% of patients are excluded under this rule. Ultimately, 224 patients were included, which we believe does not affect the generalizability of the study findings. Clinicopathological data and immunotherapy response data were abstracted from the electronic medical record. Baseline demographics, including age, gender, clinical stage, therapeutic schedule, etc., can be found in Table 1 and Table S1. Histological results and 114 peripheral blood biomarkers were recorded (Table S2).

**Table 1:** Demographics and clinical characteristics of patients

Characteristics	No. of patients	%
Age, median (range, year)	64 (32–86)	
*Gender*		
Male	173	77.2
Female	51	22.8
*Histology*		
Squamous	110	49.1
Adenocarcinoma	114	50.9
*Clinical stage*		
I–III	62	27.7
IV	144	64.3
NOS	18	8.0
*Tumor stage*		
T0–2	80	35.7
T3–4	72	32.1
Tx	72	32.1
*Node stage*		
N0–1	37	16.5
N2–3	118	52.7
Nx	69	30.8
*Metastasis stage*		
M0	67	29.9
M1	142	63.4
Mx	15	6.7
*Differentiation*		
Moderate and moderate-poor	55	24.6
Poor	96	42.9
NOS	73	32.6
*Therapeutic regimen*		
Immunotherapy	165	73.7
Immunotherapy + targeted therapy	59	26.3
*Previous treatment regimens*		
Chemotherapy or radiotherapy	115	51.3
Never before	109	48.7
*Therapeutic response*		
Partial response (PR)	260	16.2
Stable disease (SD)	976	60.8
Progressive disease (PD)	369	23.0

### Machine-learning models for therapeutic responses

We categorize the potential response to therapy as partial response (PR), stable disease (SD), or progressive disease (PD) through supervised three-way classification. None of the patients achieved complete response (CR) in our cohort. 224 patients had a total of 1605 treatment cycles, and these 1605 treatment cycles were split at random into a training set (*n* = 1124) and a test set (*n* = 321) by program automatic partition. Various classification machine-learning algorithms were used, including logistic regression (LR), random forest (RF), eXtreme gradient boosting (XGBoost), and light gradient boosting machine (LightGBM) models. Every machine -learning model divides the data into three folds, and is trained and validated repeatedly. The performance was assessed based on area under the curve (AUC) values and the confusion matrix, which measured accuracy, precision, recall, and f1 score. Details of computational formula are in Supplementary Materials.

### Statistical analysis

The duration between the beginning of immunotherapy and either PD or death was referred to as PFS. Patients who did not experience PD had their data censored during their last disease assessment scan. The Kaplan–Meier method was utilized to determine event-time distributions, which were then compared using the log-rank test. All *p* values are considered two-sided, and confidence intervals are at the 95% level. Differences in response and clinical benefit between groups with different biomarkers were compared using a two-sided t test. A *p* value of less than 0.05 was deemed statistically significant.

## Results

### Patient demographics and disease characteristics

The 224 NSCLC patients’ clinical characteristics were outlined in Table 1, which revealed that the median age was 64 years and 77.2% were male. Of the patients, 49.1% had squamous cell carcinoma (SCC) and 50.9% had adenocarcinoma (AC). Concurrent targeted therapy was given to 26.3% of patients, with 48.7% receiving it as their first-line treatment. The treatment regimen involved Tislelizumab for 28.6%, Sintilimab for 27.7%, Camrelizumab for 23.7%, Cadonilimab for 5.4%, Pembrolizumab for 5.4%, Serplulimab for 4.0%, while others received Nivolumab, Penpulimab, Toripalimab, or Zimberelimab (Figure S1). Pathology tests occurred before immunotherapy and blood biomarkers were conducted before each treatment cycle. The study assessed the therapeutic response of each treatment cycle, which classified the outcomes as PR, SD, or PD.

### Predictive capacity of PD-L1 and TMB

Pathological examination has long been considered the primary method for identifying tumors and guiding treatment, and PD-L1 and TMB in tumor tissue have been widely studied [9–12]. Hence, our research first investigated the histological outcomes of PD-L1 and TMB. From 224 patients, 113 of them had PD-L1 pathology results and 40 had TMB results. To create two comparable-sized groups, we classified patients based on their PD-L1 (low/high) and TMB (low/high) levels, using the median as the threshold for both variables. The Kaplan–Meier survival curves revealed that the group with PD-L1 TPS ≥ 10% had a significantly longer median PFS (mPFS) than the group with PD-L1 TPS < 10% (510 vs. 180 days, *p* = 0.0033, Figure S2A). During the patient’s subsequent treatment, it was noticed that individuals with a PD-L1 TPS of 10% or more received notable clinical benefits (*p* < 0.001, Figure S2C). Due to the limited availability of 40 TMB data, we found no statistical difference in mPFS (Figure S2B). In the case of the TMB-H group, the probability of achieving PR was 13% compared to only 4% for the TMB-L group, and it had a lesser chance of experiencing PD (17 vs. 33%) (Figure S2D).

### Therapeutic responses predicted by machine-learning models

**Fig. 1:**
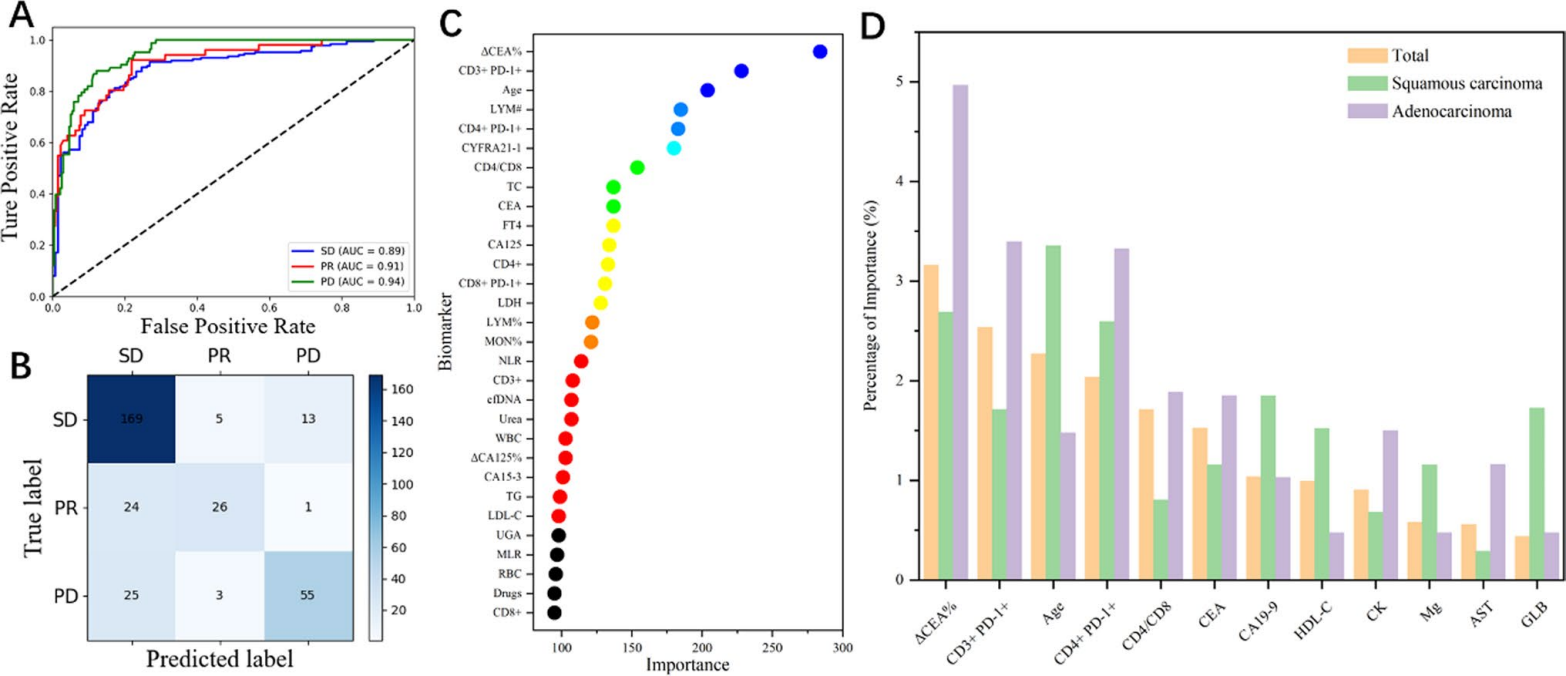
Prediction of therapeutic responses (PD, SD, and PR) through clinical features and peripheral blood biomarkers by lightGBM model. **A** Receiver operating characteristic (ROC) curves of prediction model in the validation cohort. **B** Confusion matrix of prediction model in the validation cohort. **C** Top 30 important biomarkers from the lightGBM model. **D** Important biomarkers that differ significantly between SCC and AC patients

From above, only half demonstrated PD-L1 pathological outcomes, with a mere 17.9% displaying TMB genetic test outcomes. In addition, just 14.3% individuals received both results. Consequently, accessible biomarkers like hematological biomarkers are crucial. We analyzed 11 clinical features and 114 hematological biomarkers to identify excellent prognostic biomarkers using four models, including LR, RF, XGBoost, and LightGBM (see Fig. 1 and Figure S3–S5). Each model’s performance was assessed by accuracy, precision, recall, AUC, and confusion matrices (shown in Table S3). LightGBM emerged as the top performer with an accuracy rate of 79.75%, precision rate of 81.64%, and recall rate of 70.79%. The f1 score, which takes into account precision and recall, was 74.58%. The AUCs for therapeutic responses (PD, SD, and PR) in the validation group are high with values of 0.94, 0.89, and 0.91 respectively (Fig. 1A). In addition, Fig. 1C depicts the particularly significant immune cell subsets and serum markers.

Then, Fig. 1D entails conducting distinct analyses on patients diagnosed with SCC and AC. Based on statistical evidence, the biomarkers that display notable variations are ΔCEA%, CD3^+^ PD-1^+^, age, CD4^+^ PD-1^+^, CD4/CD8, and others. Among these, ΔCEA%, CD3^+^PD-1^+^, CD4^+^PD-1^+^, and CD4/CD8 hold greater significance in forecasting the prognosis of AC patients, while age proves to be more crucial for SCC patients.

### Analysis of peripheral immune cell subsets

**Fig. 2:**
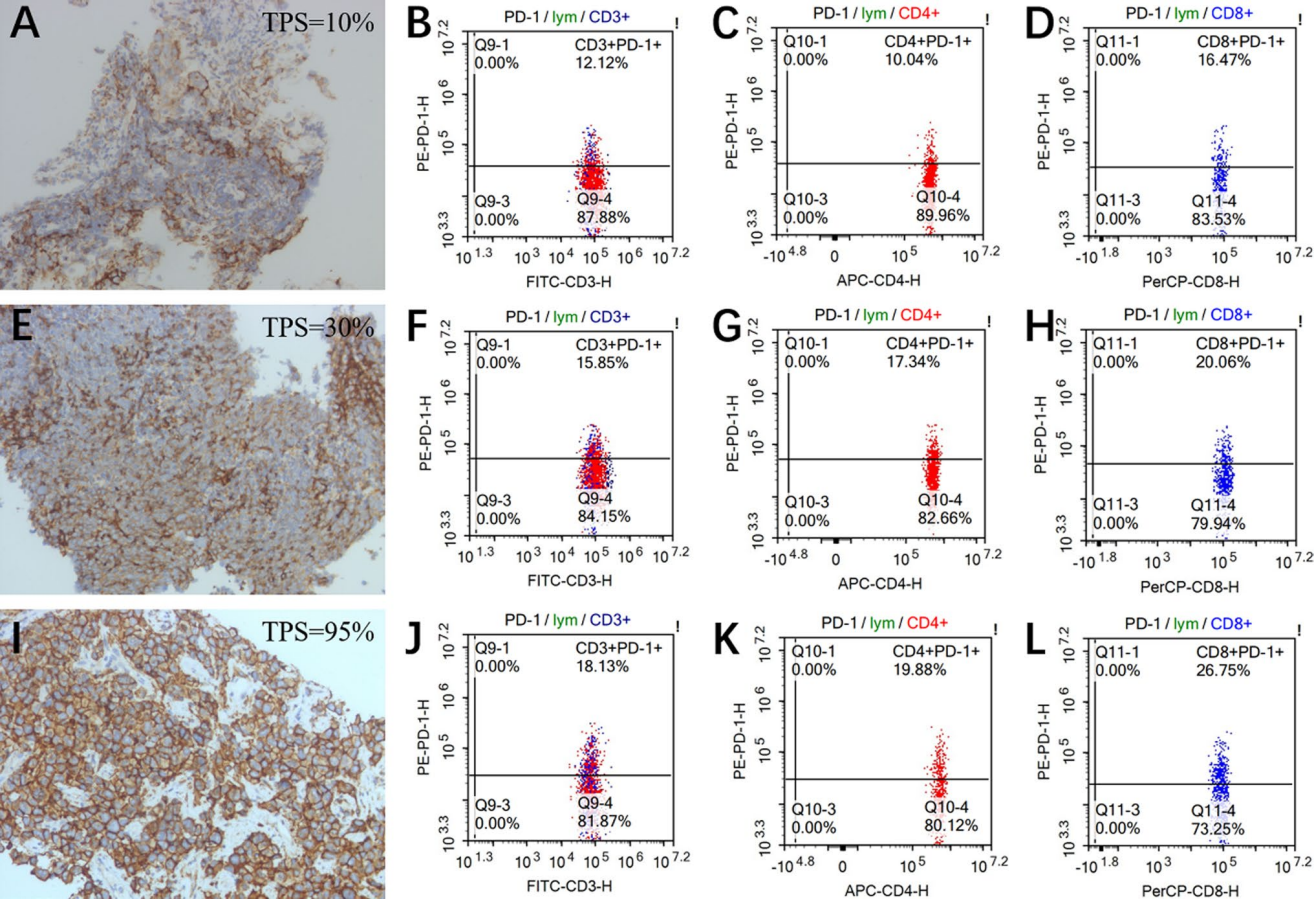
Relationship between PD-L1 levels in tumor tissue and PD-1 levels in peripheral blood. **A–D** PD-L1 expression in tumor tissue with TPS of 10% (**A**), and corresponding PD-1 expression on CD3^+^ T cells (**B**), CD4^+^ T cells (**C**), and CD8^+^ T cells (**D**) in peripheral blood during the same period. **E–H** PD-L1 expression in tumor tissue with TPS of 30% (**E**), and corresponding PD-1 expression on CD3^+^ T cells (**F**), CD4^+^ T cells (**G**) and CD8^+^ T cells (**H**) in peripheral blood during the same period. **I–L** PD-L1 expression in tumor tissue with TPS of 95% (**I**), and corresponding PD-1 expression on CD3^+^ T cells (**J**), CD4^+^ T cells (**K**) and CD8^+^ T cells (**L**) in peripheral blood during the same time frame. Details of flow cytometry are in Supporting Information

**Fig. 3:**
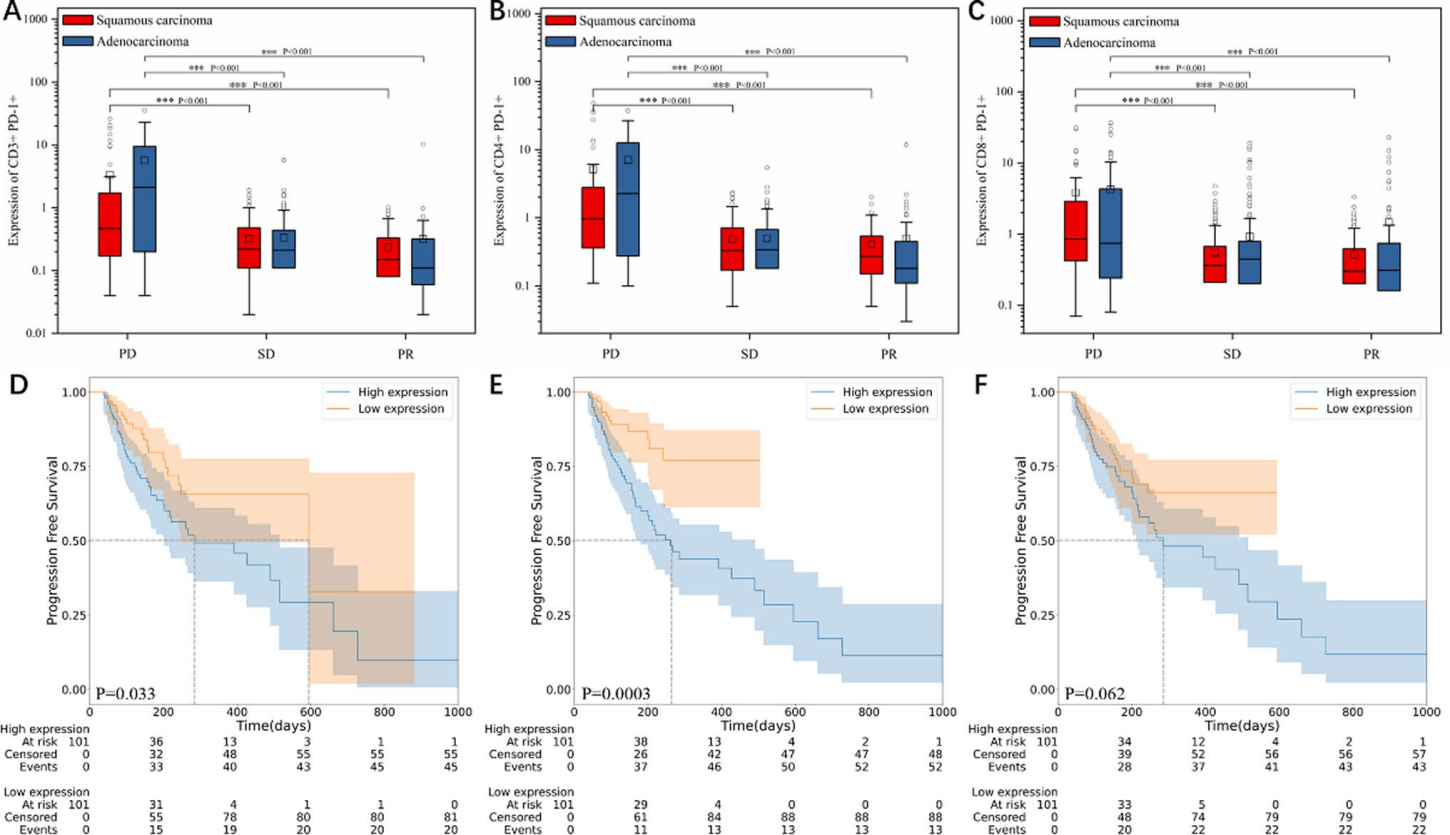
Predictive ability of PD-1 expression. **A–C** Expression of CD3^+^PD-1^+^ (**A**), CD4^+^PD-1^+^ (**B**), and CD8^+^PD-1^+^ (**C**) in PD, SD, and PR groups. **D–F** Kaplan–Meier curves for PFS of average CD3^+^PD-1^+^ (**D**), CD4^+^PD-1^+^ (**E**), and CD8^+^PD-1^+^ (**F**). The median of the expression is used as the cutoff criterion. The statistics do not include the results of the first treatment cycle

The LightGBM model indicates that immune cell subsets may be valuable prognostic predictive markers, and then we investigate the connection between these subsets and PD-L1 histological results. In Fig. 2, we noted that the tendency of PD-L1 TPS aligns with PD-1 expression in peripheral blood at the same time. For a patient has a TPS of 10%, their PD-1 expression levels of CD3^+^, CD4^+^, and CD8^+^ T cells will display 12.12, 10.04, and 16.47%, respectively. Similarly, for a patient has a TPS of 95%, their PD-1 expression levels will escalate to 18.13, 19.88, and 26.75%. After their second treatment cycle, most patients exhibit a reduction in PD-1 expression (Figure S6). This led us to explore the correlation between PD-1 expression and prognosis, and discovered that high levels of PD-1 are consistently present in the PD clade (*p* values <0.001, Fig. 3A–C). Besides, we observed a significant difference in the prognosis of AC patients for CD3^+^PD-1^+^ and CD4^+^PD-1^+^, which corroborates the results from our model in Fig. 1D. Furthermore, our research revealed that CD3^+^PD-1^+^, CD4^+^PD-1^+^, and CD8^+^PD-1^+^ with low expression had a longer mPFS, as evidenced in Fig. 3D–F.

In addition to PD-1 expression, our study highlights the importance of several factors, including CD4/CD8, lymphocyte percentage (LYM%), monocyte percentage (MON%), white blood cell count (WBC), neutrophil-to-lymphocyte ratio (NLR), and monocyte-to-lymphocyte ratio (MLR). A significant increase in CD4/CD8, LYM%, and MON% expression in the PR group, while there was a decrease in WBC, NLR, and MLR expression (Figure S7). Our analysis of PFS showed similar trends, with higher expression of CD4/CD8 and LYM% indicating longer mPFS, and lower expression of NLR and MLR indicating extended mPFS (Figure S8).

### Role of serum markers

**Fig. 4:**
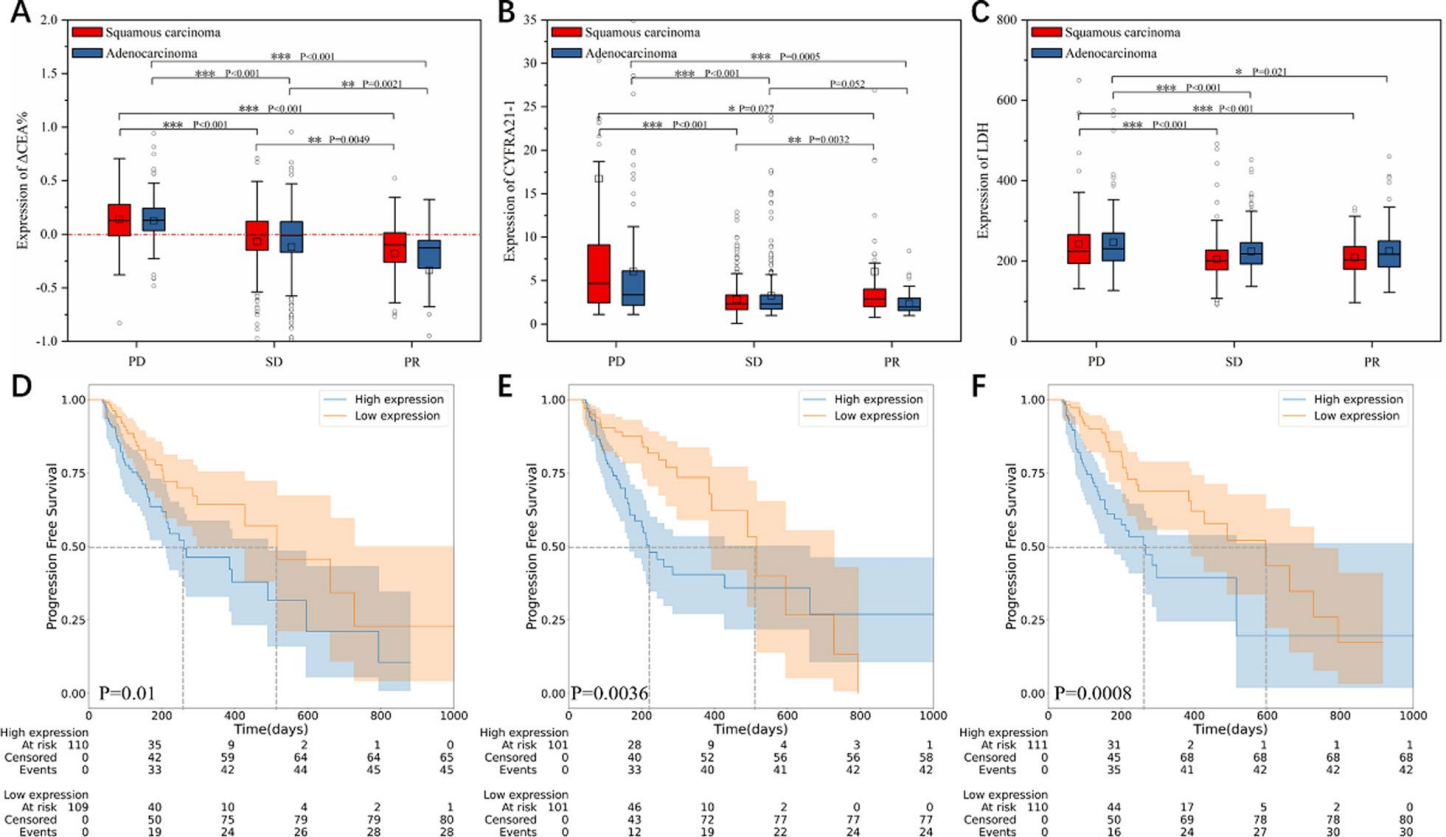
Analysis of serum immunological biomarkers. **A–C** Expression of ΔCEA% (**A**), CYFRA21-1 (**B**), and LDH (**C**) in PD, SD, and PR groups. **D–F** Kaplan–Meier curves for PFS of average ΔCEA% (**D**), CYFRA21-1 (**E**) and LDH (**F**). The median of the expression is used as the cutoff criterion. The statistics do not include the results of the first treatment cycle

The lightGBM model also provided valuable insights into serum biochemical and immunological biomarkers. Notably, the change rate of carcinoembryonic antigen (ΔCEA%) exhibited significant differences between the PD, SD, and PR groups (Fig. 4A). Patients in ΔCEA% < 0 group had a longer mPFS of 510 days compared to 250 days for other groups, with a *p* value of 0.01 (Fig. 4D). In addition, patients with cytokeratin 19 fragment (CYFRA21-1) levels below 2.65 ng/ml had a longer mPFS of 510 versus 220 days for those with levels ≥2.65 ng/ml, with a *p* value of 0.0036 (see Fig. 4E). Furthermore, an average LDH expression below 220 U/L corresponded to a longer mPFS of 600 days compared to 270 days for others (*p* = 0.0008, Fig. 4F). Although the model identified other biomarkers such as total cholesterol (TC), free thyroxine (FT4), CA125, urea, etc., we failed to establish any significant relationship between these markers and therapeutic response after conducting an analysis (Figure S9).

### Study of clinical characteristics

We then examined the impact of tumor type, immunotherapy drugs, and age. By the PFS with different tumor types, we discovered no significant difference in SCC and AC (Figure S10A). As can be seen from the longer duration, patients with SCC have a better prognosis. Furthermore, we evaluated three commonly used immunotherapy drugs and found no significant difference in their effectiveness (as demonstrated in Figure S10B). We also divided the patients into SCC and AC and still found no significant variation from different drugs (Figure S11). Subsequently, we divided our patients into two groups based on age (i.e., those younger and older than 60 years) and discovered no significant difference in PFS (Figure S12). Fortunately, the effect of age can be seen when we discuss SCC and AC separately. The SCC patients younger than 60 years had statistically longer mPFS, but the AC patients had no effect of age (Figure S10C, D).

### Combined diagnosis by detection panel consisting of multi-biomarkers

**Fig. 5:**
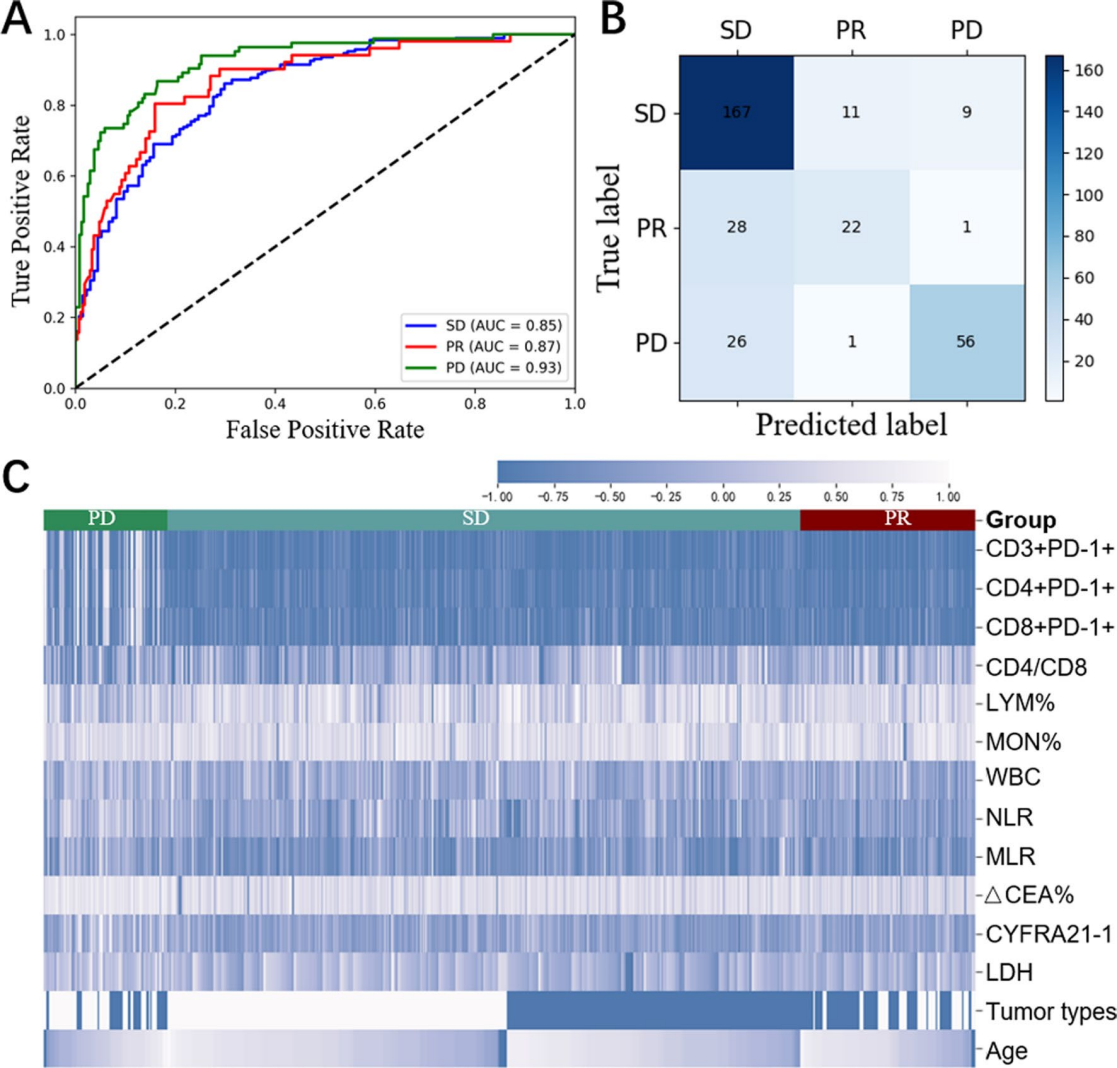
Prediction of therapeutic responses through the 14 crucial markers. **A** ROC curves of a prediction model in the validation cohort. **B** Confusion matrix of prediction model in the validation cohort. **C** Heat map of median arcsinh-transformed biomarker expression normalized per batch to a mean of 0. White indicates relative marker over-expression, and blue indicates relative under-expression. Bars at the top of the heat map represent individual samples from PD (*green*), SD (*light green*), and PR (*dark red*). In the representation of tumor types, white indicates AC and blue indicates SCC. Each column represents one patient sample from one-time point. The statistics do not include the results of the first treatment cycle

After utilizing the lightGBM model and subsequent analysis, we have successfully pinpointed attainable 14 important biomarkers to form our unique detection panel that can predict therapeutic responses accurately. These include CD3^+^PD-1^+^, CD4^+^PD-1^+^, CD8^+^PD-1^+^, CD4/CD8, LYM%, MON%, WBC, NLR, MLR, ΔCEA%, CYFRA21-1, LDH, tumor types and age. In addition, we evaluated the diagnostic capability of this detection panel. Predicted performance indicate an accuracy, precision, recall, and f1 score of 76.3, 75.0, 66.6, and 69.6%, respectively. The AUCs for predicting PD, SD, and PR were 0.85, 0.87, and 0.93, as illustrated in Fig. 5A. The heat map in Fig. 5C shows differences in the expression of these biomarkers between different groups. Among them, biomarkers such as CD3^+^PD-1^+^, CD4^+^PD-1^+^, CD8^+^PD-1^+^, WBC, NLR, MLR, ΔCEA%, CYFRA21-1, and LDH was significantly over-expressed in the PD group, while CD4/CD8, LYM%, and MON% are highly expressed in the PR group.

## Discussion

Although ICIs have been successful in clinical trials for NSCLC, individual differences and tumor microenvironments make it possible for not all patients to achieve long-term OS. Biomarkers derived from tumor tissue are critical, but our results reveal that only 14.3% of NSCLC patients have both PD-L1 and TMB results. Alternatively, hematological biomarkers are non-invasive, reproducible, relatively affordable, and more importantly, they can be continuously and dynamically monitored. We utilized four machine-learning models to evaluate 11 clinical features and 114 peripheral blood biomarkers for predicting responses to anti-PD-1 immunotherapy. The lightGBM model demonstrated an impressive accuracy rate of 79.75%, and 14 significant biomarkers were identified. These filtered biomarkers were then utilized to predict responses with an accuracy rate of 76.3%.

The success of immunotherapy is largely dependent on the activation of “silent” lymphocytes that can permanently and effectively eliminate tumor cells. The number and various subsets of lymphocytes are crucial factors in determining the efficacy and prognosis of immunotherapy. From a set of 14 crucial biomarkers, immune cell subsets can provide valuable insights into the anti-PD-1 response. By examining PD-L1 levels in tumor tissue and PD-1 in the blood, we observed a similar trend in their changes. This similarity indicates that the PD-1 level is just as important a predictor of the prognosis as PD-L1. During subsequent anti-PD-1 immunotherapy, almost all patients experience a decrease in PD-1 expression, and the PD group has higher PD-1 expression. This is because anti-PD-1 ICIs inhibit the expression of PD-1 in T cells effectively. Our research also explored other immune cell subsets and found that the PR group had higher levels of CD4/CD8, LYM%, and MON%, and lower levels of WBC, NLR, and MLR. Higher expression of CD4/CD8 and LYM%, and lower expression of NLR and MLR, reveal longer mPFS. CD4^+^ T cells can promote and regulate the initiation, migration potential and killing activity of cytotoxic T lymphocytes, while CD8^+^ T cells have cytotoxic and immunosuppressive effects. CD4/CD8 is a reliable indicator positively linked with therapeutic responses [23, 24]. LYM is an important component of the body’s immunity and plays an important role in the anti-tumor process. A decrease in LYM% is associated with a worse prognosis. Furthermore, ICIs therapy mainly uses T lymphocytes to kill tumors, and a decrease in the number of T lymphocytes, due to inflammation, may reduce the effect of ICI therapy. Consequently, high levels of NLR and MLR, which indicate systemic inflammation, may suggest the PD stage and shorter mPFS. Neutrophils are not released from the bone marrow until they mature, but in the context of inflammation, the neutrophil precursors, myelocytes and promyelocytes, may be released. The prognostic and predictive usefulness of circulating neutrophils is apparent as an independent measure or as part of the NLR [25, 26]. Monocytes in the circulation give rise to macrophages that reside in tissue, and tumor-associated macrophages (TAMs) release factors that promote new blood vessel growth, aiding cancer spread, which make the MLR a dependable predictive biomarker for tumor progression [27].

Serum biomarkers, such as ΔCEA% and CYFRA21-1, are important for diagnosing and predicting the severity of lung cancer. CEA is a protein in the blood that is commonly used to detect lung cancer, and can also predict the outcome of treatment [7]. In our study, patients in the PR group tended to have lower levels of CEA than in their previous treatment cycle, whereas levels remained stable in the SD group and increased in the PD group. Patients with a ΔCEA% value less than 0 had a longer mPFS than those with a value greater than or equal to 0. CYFRA21-1 is abundant in the pulmonary tissue, and correlated with the tumor size, lymph node status, and disease stage [28]. Elevated levels of CYFRA21-1 may indicate that the patient is in the PD stage, and those with CYFRA21-1 levels <2.65 ng/ml experienced longer mPFS than those with ≥2.65 ng/ml. In addition, LDH, as a serum biochemical marker, is released into the bloodstream when tumor cells multiply abnormally, indicating rapid tumor growth [29]. High expression therefore indicates that the tumor is expanding quickly, and patients with an expression level above 220 U/L are likely to have poor PFS.

Our research showed no statistical difference in their PFS for patients with SCC and AC, but we found that SCC patients have a better prognosis with longer durations. Notably, AC patients showed more noticeable changes in CD3^+^PD-1^+^, CD4^+^PD-1^+^, CD4/CD8, and ΔCEA%, making it crucial to distinguish the course of the disease compared to SCC. Interestingly, the efficacy of various immunotherapy drugs was similar, implying that patients can choose high-quality and inexpensive anti-PD-1 immunotherapy drugs. Our research also yielded promising results regarding patient age and prognosis. The SCC patients younger than 60 years of age had longer mPFS, but no statistical pattern was observed in AC patients. These differences between SCC and AC may be attributed to the heterogeneity of different cancer types, and further exploration of the specific mechanism is needed in future work.

In summary, we have developed a highly accurate model for predicting the success of anti-PD-1 immunotherapy in NSCLC patients. By detection panel consisting of 14 crucial biomarkers, we achieved a prediction accuracy of 76.3%. Our research identified a correlation between histological PD-L1 TPS and hematological PD-1 expression. Notably, in follow-up dynamic monitoring, a higher level of PD-1 is associated with a poor prognosis. Furthermore, patients with a higher CD4/CD8 and LYM%, and lower NLR and MLR levels, have a higher chance of achieving PR and longer mPFS. We also identified LDH, ΔCEA%, and CYFRA21-1 as valuable predictors. These findings form our exclusive detection panel for NSCLC patients to be able to develop personalized treatment regimens and avoid over- and under-treatment. And because hematological biomarkers can be monitored in real time, our panel results can be used to dynamically adjust the current treatment regimen.

### Supplementary Information

Below is the link to the electronic supplementary material.

Supplementary file The online version contains supplementary material available at https://doi.org/ (PDF 1814 KB)

## Data Availability

The datasets generated during the present study are available from the corresponding author upon reasonable request.
