# Supplementary material for: Variation of peripheral blood-based biomarkers for response of anti-PD-1 immunotherapy in non-small-cell lung cancer
Source: Clin Transl Oncol. 2024 Mar 7;26(8):1934–43. doi: 10.1007/s12094-024-03416-5 (PMC11249409; doi:10.1007/s12094-024-03416-5)
Supplement: Supplementary file 1 — Supplementary file The online version contains supplementary material available at https://doi.org/ (PDF 1814 KB) [file 12094_2024_3416_MOESM1_ESM.pdf]

Supplementary Materials for

Variation of Peripheral Blood-based Biomarkers for Response of Anti-PD-1 Immunotherapy in Non-Small-Cell Lung Cancer

Xiaoming Wang, Dayu Chen, Yuyan Ma, Dongping Mo, Feng Yan*

Department of Clinical Laboratory, Nanjing Medical University Affiliated Cancer Hospital & Jiangsu Cancer Hospital & Jiangsu Institute of Cancer Research; Nanjing, 210009; Jiangsu, China

*Corresponding author: Feng Yan, Department of Clinical Laboratory, Nanjing Medical University Affiliated Cancer Hospital & Jiangsu Cancer Hospital & Jiangsu Institute of Cancer Research, Baizi Ting No.42, Nanjing, Jiangsu 210009, China. Tel: +86-25-83286401; E-mail: yanfeng@jszlyy.com.cn

Methods 1. Flow Cytometry

Methods 2. Computational Formula of accuracy, precision, recall, and f1 score

Methods 3. Treatment and Response Assessment

Figure S1. Immunotherapy drugs from 224 patients.

Figure S2. Predictive capacity of PD-L1 and TMB. (**A, B**) Kaplan–Meier curves for PFS of PD-L1 (**A**) and TMB (**B**). (**C, D**) Probability of clinical responses predicted by PD-L1 (**C**) and TMB (**D**).

Figure S3. Prediction of therapeutic responses (PD, SD, and PR) through clinical features and peripheral blood biomarkers by logistic regression (LR) model. (**A**) ROC curves of prediction model in the validation cohort. (**B**) Confusion matrix of prediction model in the validation cohort.

Figure S4. Prediction of therapeutic responses (PD, SD, and PR) through clinical features and peripheral blood biomarkers by random forest (RF). (**A**) ROC curves of prediction model in the validation cohort. (**B**) Confusion matrix of prediction model in the validation cohort.

Figure S5. Prediction of therapeutic responses (PD, SD, and PR) through clinical features and peripheral blood biomarkers by XGBoost. (**A**) ROC curves of prediction model in the validation cohort. (**B**) Confusion matrix of prediction model in the validation cohort.

Figure S6. Dynamic monitoring of PD-1 expression. Expression of $CD3^{+}PD\text{-}1^{+}$ (A), $CD4^{+}PD\text{-}1^{+}$ (B), and $CD8^{+}PD\text{-}1^{+}$ (C) at different treatment cycle.

Figure S7. Role of certain immune cells. Expression of CD4/CD8 (**A**), LYM% (**B**), MON% (**C**), WBC (**D**), NLR (**E**) and MLR (**F**) in PD, SD, and PR groups.

Figure S8. Role of certain immune cells. Kaplan–Meier curves for PFS of average expression of CD4/CD8 (**A**), LYM% (**B**), MON% (**C**), WBC (**D**), NLR (**E**) and MLR (**F**) throughout treatment. The median of the expression is used as the cutoff criterion. The statistics do not include the results of the first treatment cycle.

Figure S9. Role of certain serum biochemical and immunological markers. Expression of TC (**A**), FT4 (**B**), CA125 (**C**), Urea (**D**), CA15-3 (**E**), TG (**F**), LDL-C (**G**) and UGA (**H**) in PD, SD, and PR groups.

Figure S10. Effect of clinical characteristics. Kaplan–Meier curves for PFS of different tumor types (**A**), immunotherapy drugs (**B**), age of SCC patients (**C**), and AC patients (**D**). The 60 years of age is used as the cutoff criterion.

Figure S11. Effect of patient's age and different immunotherapy drugs. (**A**) Kaplan–Meier curves for PFS of ages of patients. (**B**) Probability of clinical responses treated by Sintilimab, Tislelizumab, and Camrelizumab.

Figure S12. Effect of patient's age. Kaplan–Meier curves for PFS of ages of patients. The 60 years of age is used as the cutoff criterion.

Table S1. Clinical characteristics of patients.

Table S2. The 114 biomarkers in peripheral blood.

Table S3. Performances of LR, RF, XGBoost, and LightGBM models.

Methods 1. Flow Cytometry

Peripheral blood mononuclear cells (PBMCs) were isolated and treated with antibodies after eliminating the red blood cells. Subsequently, the samples were assessed on an Agilent NovoCyte flow cytometer. To minimize inconsistencies in optics that may arise from day to day, the flow cytometer was calibrated using fluorescence beads before each experiment. This calibration process guaranteed that the measurement conditions were consistent across different samples. The operating procedures of the test kit are strictly following the instructions provided by Jiangxi Ceger Biotechnology Co., LTD.

Methods 2. Computational Formula of accuracy, precision, recall, and f1 score

Accuracy means the percentage of samples that make the correct guess among all available samples.

$$\text{Accuracy} = \frac{\text{TP} + \text{TN}}{\text{TP} + \text{TN} + \text{FP} + \text{FN}} \times 100\%$$

Precision is defined as a corrective prediction in the positive class of samples.

$$\text{Precision} = \frac{\text{TP}}{\text{TP} + \text{FP}} \times 100\%$$

The recall is indicated how successfully positive states were predicted.

$$\text{Recall} = \frac{\text{TP}}{\text{TP} + \text{FN}} \times 100\%$$

F1 score takes into account both the precision and recall of classification model.

$$\text{f1 score} = 2 \times \frac{\text{Precision} \times \text{Recall}}{\text{Precision} + \text{Recall}}$$

In equations, a true positive (TP) occurs when the algorithm accurately predicts the patient's classification, whereas a false positive (FP) arises when the algorithm inaccurately predicts a patient’s classification. A true negative (TN) occurs when the algorithm correctly predicts that a patient does not belong to a certain classification, and a false negative (FN) arises when the algorithm fails to predict that a patient belongs to a certain classification.

Methods 3. Treatment and Response Assessment

To evaluate the efficacy of treatment, computed tomography or magnetic resonance imaging were utilized. The Response Evaluation Criteria in Solid Tumors (RECIST ver 1.1) was used to assess tumor response, which was then classified into one of four categories: complete response (CR), partial response (PR), stable disease (SD), or progressive disease (PD).

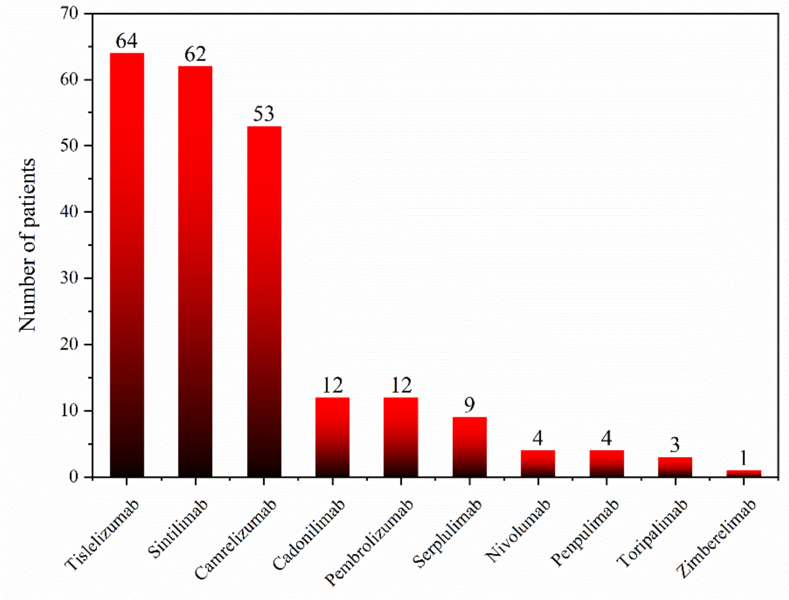

Figure S1. Immunotherapy drugs from 224 patients.

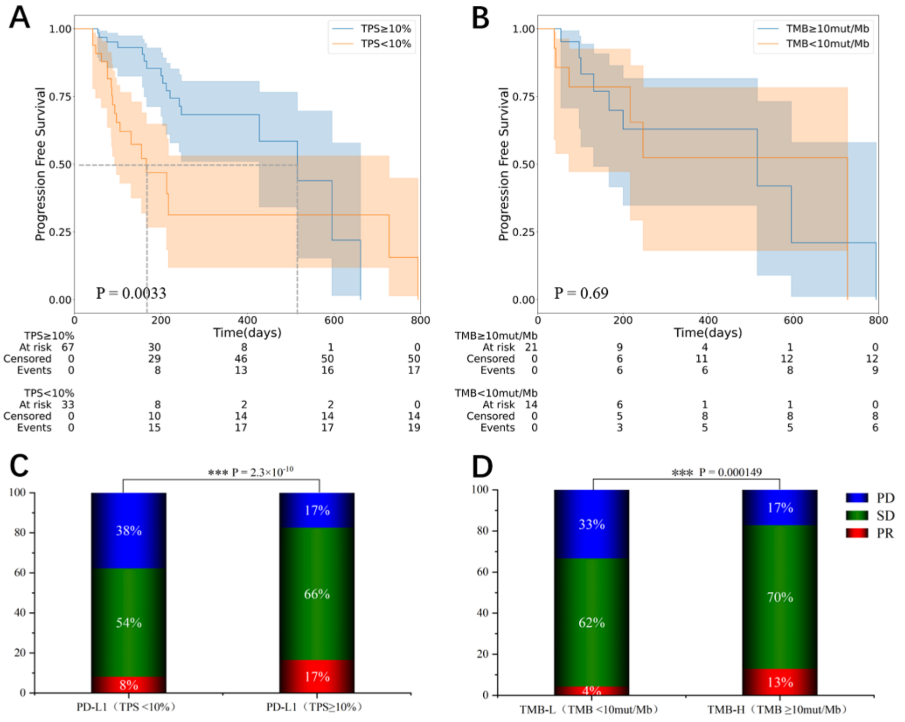

Figure S2. Predictive capacity of PD-L1 and TMB. (**A, B**) Kaplan–Meier curves for PFS of PD-L1 (**A**) and TMB (**B**). (**C, D**) Probability of clinical responses predicted by PD-L1 (**C**) and TMB (**D**).

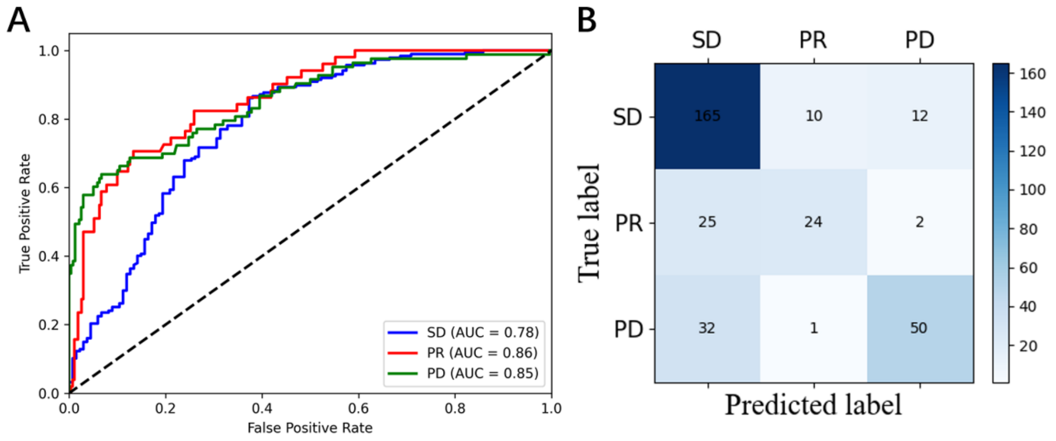

Figure S3. Prediction of therapeutic responses (PD, SD, and PR) through clinical features and peripheral blood biomarkers by logistic regression (LR) model. (**A**) ROC curves of prediction model in the validation cohort. (**B**) Confusion matrix of prediction model in the validation cohort.

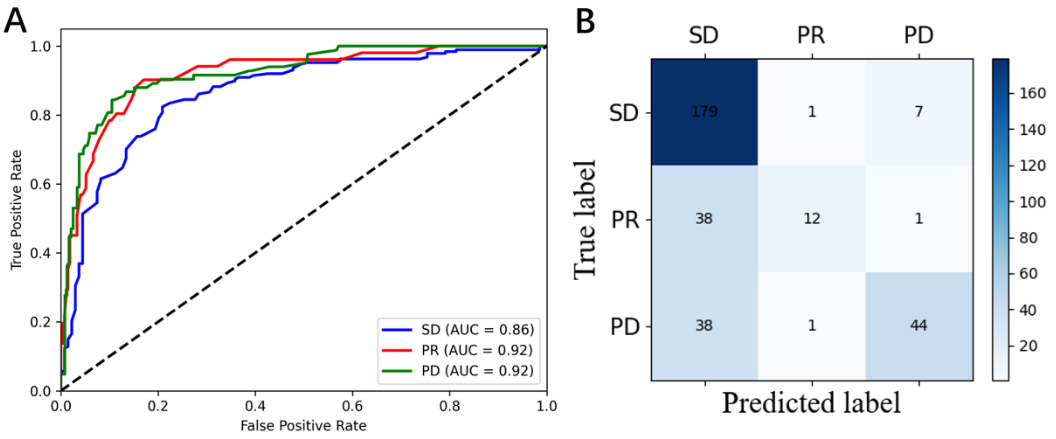

Figure S4. Prediction of therapeutic responses (PD, SD, and PR) through clinical features and peripheral blood biomarkers by random forest (RF). (**A**) ROC curves of prediction model in the validation cohort. (**B**) Confusion matrix of prediction model in the validation cohort.

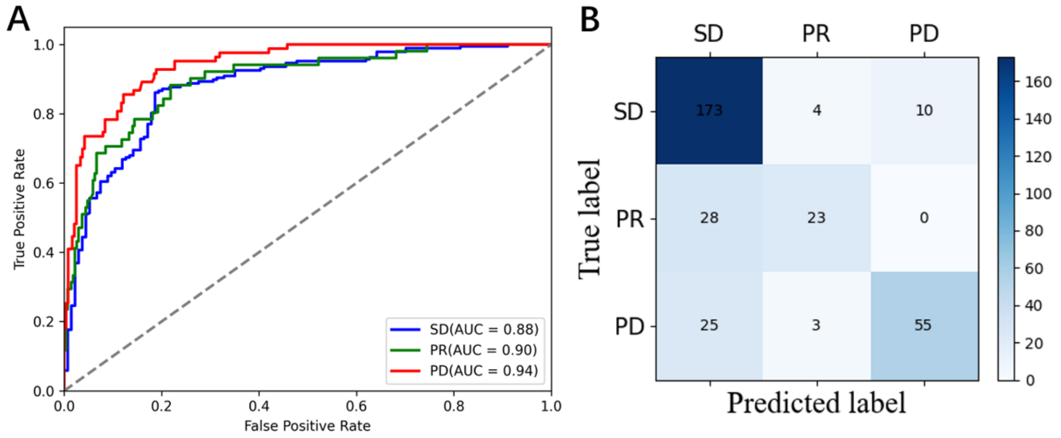

Figure S5. Prediction of therapeutic responses (PD, SD, and PR) through clinical features and peripheral blood biomarkers by XGBoost. (**A**) ROC curves of prediction model in the validation cohort. (**B**) Confusion matrix of prediction model in the validation cohort.

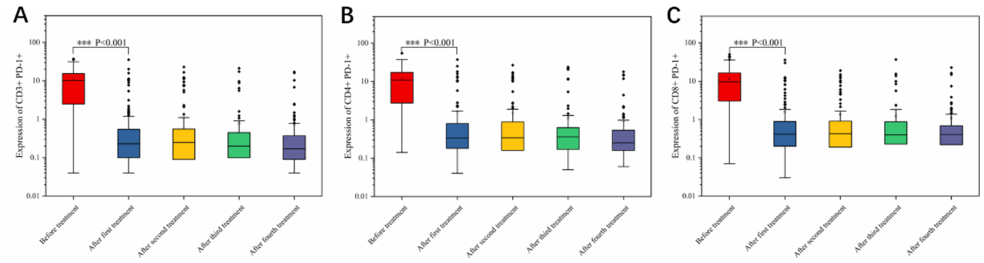

Figure S6. Dynamic monitoring of PD-1 expression. Expression of CD3^{+}PD-1^{+} (**A**), CD4^{+}PD-1^{+} (**B**), and CD8^{+}PD-1^{+} (**C**) at different treatment cycle.

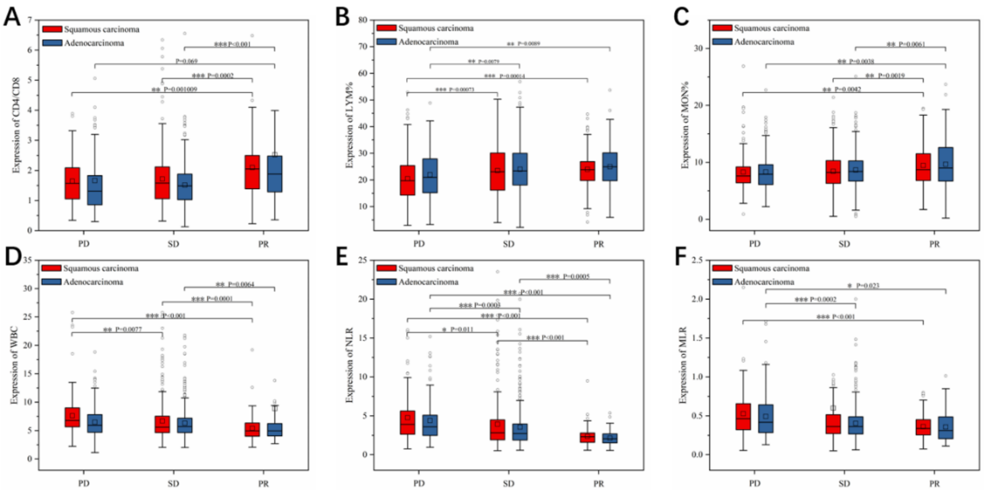

Figure S7. Role of certain immune cells. Expression of CD4/CD8 (**A**), LYM% (**B**), MON% (**C**), WBC (**D**), NLR (**E**) and MLR (**F**) in PD, SD, and PR groups.

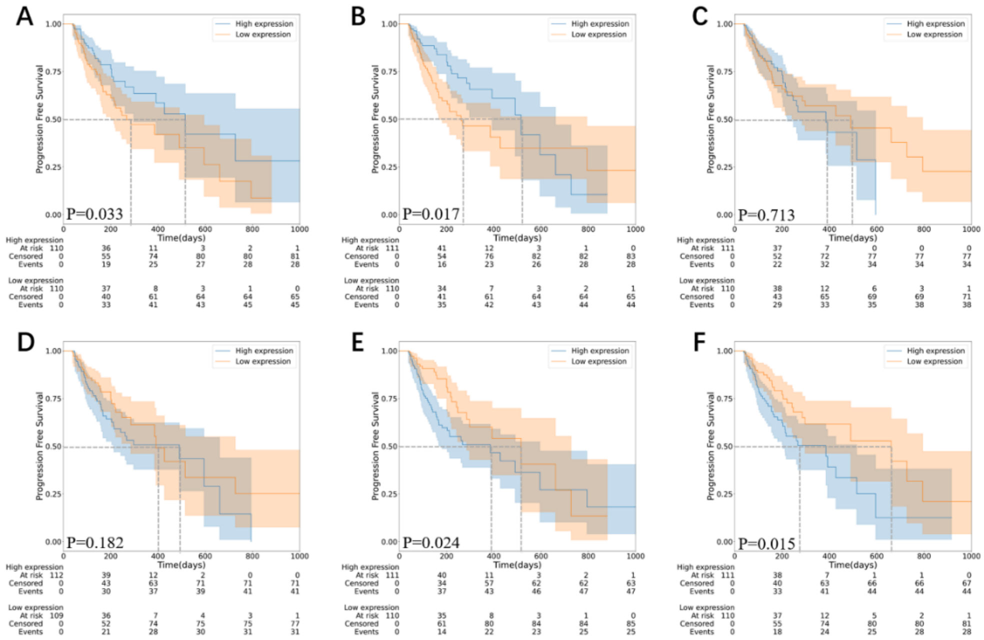

Figure S8. Role of certain immune cells. Kaplan–Meier curves for PFS of average expression of CD4/CD8 (**A**), LYM% (**B**), MON% (**C**), WBC (**D**), NLR (**E**) and MLR (**F**) throughout treatment. The median of the expression is used as the cutoff criterion. The statistics do not include the results of the first treatment cycle.

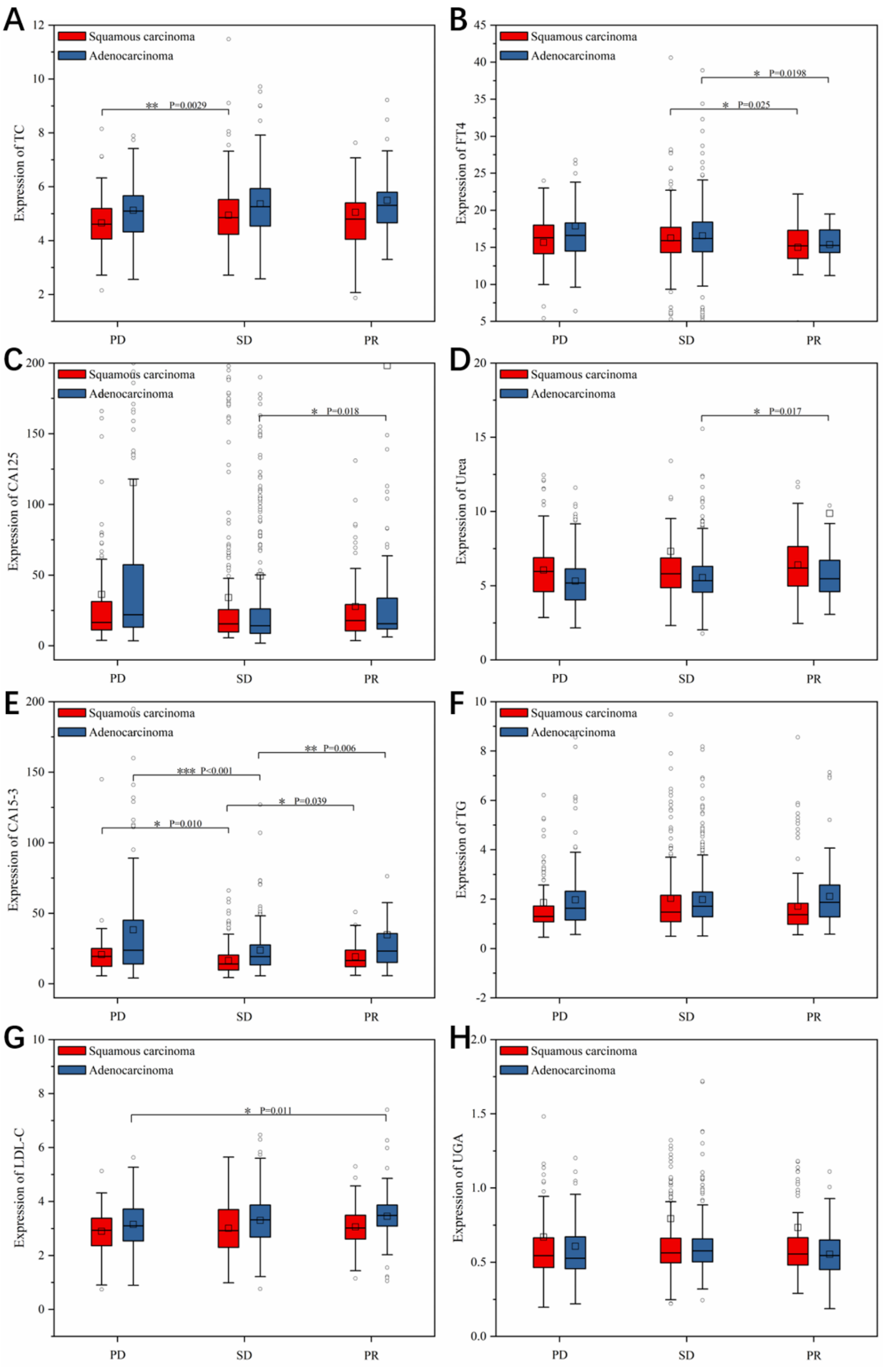

Figure S9. Role of certain serum biochemical and immunological markers. Expression of TC (**A**), FT4 (**B**), CA125 (**C**), Urea (**D**), CA15-3 (**E**), TG (**F**), LDL-C (**G**) and UGA (**H**) in PD, SD, and PR groups.

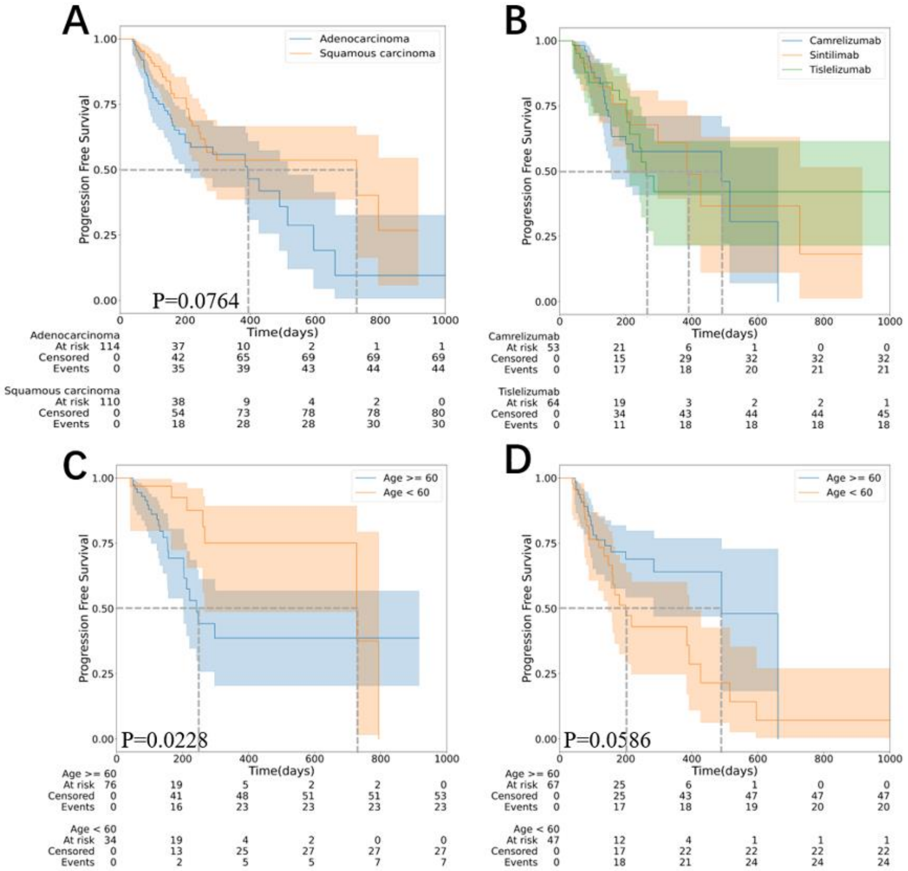

Figure S10. Effect of clinical characteristics. Kaplan–Meier curves for PFS of different tumor types (**A**), immunotherapy drugs (**B**), age of SCC patients (**C**), and AC patients (**D**). The 60 years of age is used as the cutoff criterion.

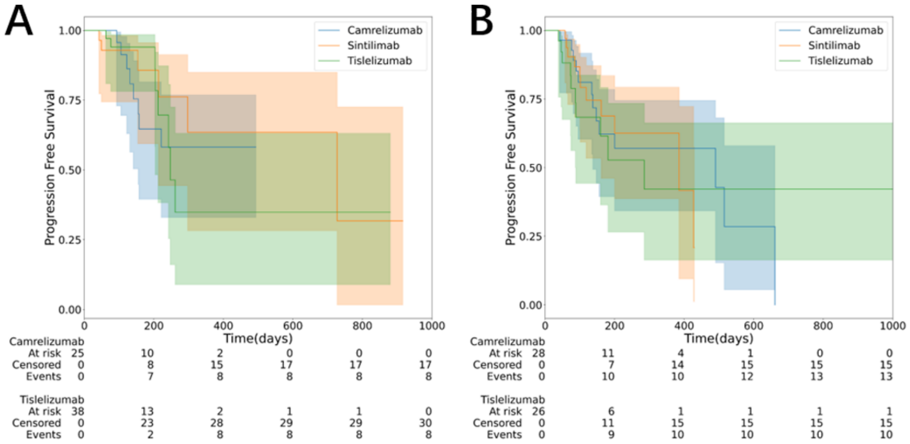

Figure S11. Effect of patient's age and different immunotherapy drugs. (**A**) Kaplan–Meier curves for PFS of ages of patients. (**B**) Probability of clinical responses treated by Sintilimab, Tislelizumab, and Camrelizumab.

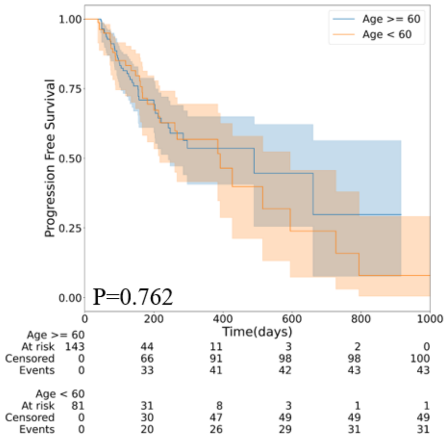

Figure S12. Effect of patient's age. Kaplan–Meier curves for PFS of ages of patients. The 60 years of age is used as the cutoff criterion.

Table S1. Clinical characteristics of patients.

	Clinical characteristics	Numeralization	Data sources
1	Age	–	Medical history
2	Gender	Male: 1; Female: 2	Medical history
3	Tumor stage (T)	0; 1; 2; 3; 4; x	Medical history
4	Node stage (N)	0; 1; 2; 3; x	Medical history
5	Metastasis stage (M)	0; 1; x	Medical history
6	Clinical stages	1; 2; 3; 4; x	Medical history
7	Differentiated degree	Moderate: 1; Moderate-poor: 0.5; Poor: 0; x	Medical history
8	Histologic type of tumor	Squamous: 1; Adenocarcinoma: 2	Medical history
9	Combined targeting therapy or not	Yes: 1; No: 0	Medical history
10	Whether prior radiotherapy or chemotherapy	Yes: 1; No: 0	Medical history
11	Immunotherapy drugs	Tislelizumab: 2;<br>Sintilimab: 3;<br>Camrelizumab: 4;<br>Cadonilimab: 5;<br>Pembrolizumab: 6;<br>Serplulimab: 9;<br>Nivolumab: 7;<br>Penpulimab: 8;<br>Toripalimab: 12;<br>Zimberelimab: 10	Medical history

x: unknown

Table S2. The 114 biomarkers in peripheral blood.

	Biomarker name	Abbreviation	Test method	Instrument
1	Percentage of total T cell	$CD3^+$	Flow fluorescence	Novocyte
2	Percentage of help T cell	$CD4^+$	Flow fluorescence	Novocyte
3	Percentage of cytotoxic T cell	$CD8^+$	Flow fluorescence	Novocyte
4	help T cell to cytotoxic T cell ratio	CD4/CD8	Calculation method	Novocyte
5	Expression rate of PD-1 on total T cell	$CD3^+PD\text{-}1^+$	Flow fluorescence	Novocyte
6	Expression rate of PD-1 on help T cell	$CD4^+PD\text{-}1^+$	Flow fluorescence	Novocyte
7	Expression rate of PD-1 on cytotoxic T cell	$CD8^+PD\text{-}1^+$	Flow fluorescence	Novocyte
8	Percentage of double positive T cells	$CD3^+CD4^+CD8^+$	Flow fluorescence	Novocyte
9	Percentage of double negative T cells	$CD3^+CD4^-CD8^-$	Flow fluorescence	Novocyte
10	Concentration of plasma free DNA	cfDNA	Flow fluorescence	Luminex MAGPIX
11	Ratio of primitive naive cells	CD45dim	Flow fluorescence	BD FACS Canto II
12	Ratio of natural killer cell	$CD3^-CD56^+$	Flow fluorescence	BD FACS Canto II
13	Ratio of B cell	$CD19^+$	Flow fluorescence	BD FACS Canto II
14	Ratio of natural killer T cell	$CD3^+CD56^+$	Flow fluorescence	BD FACS Canto II
15	Ratio of activated T cell	$CD3^+/HLA\text{-}DR^+$	Flow fluorescence	BD FACS Canto II
16	Ratio of T_{reg} cells	$CD4^+CD25^+CD127^-$	Flow fluorescence	BD FACS Canto II
17	Ratio of T_{reg} cells	$CD4^+CD25^+$	Flow fluorescence	BD FACS Canto II
18	White blood cell	WBC	Flow cytometry	Sysmex XN
19	Hemoglobin	HGB	Flow cytometry	Sysmex XN
20	Red Blood Cell	RBC	Flow cytometry	Sysmex XN
21	Platelet	PLT	Impedance method	Sysmex XN
22	hematocrit	HCT	Total red blood cell volume	Sysmex XN
23	Percentage of neutrophil	NEU%	Calculation method	Sysmex XN
24	Percentage of lymphocyte	LYM%	Calculation method	Sysmex XN
25	Percentage of monocyte	MON%	Calculation method	Sysmex XN
26	Percentage of eosinophils	EOS%	Calculation method	Sysmex XN
27	Percentage of basophils	BAS%	Calculation method	Sysmex XN
28	Neutrophil count	NEU#	Flow cytometry	Sysmex XN
29	Lymphocyte count	LYM#	Flow cytometry	Sysmex XN
30	Monocyte count	MON#	Flow cytometry	Sysmex XN
31	Eosinophils count	EOS#	Flow cytometry	Sysmex XN
32	Basophils count	BAS#	Flow cytometry	Sysmex XN
33	Plateletocrit	PCT_1	Calculation method	Sysmex XN
34	Percentage of reticulocytes	RET%	Calculation method	Sysmex XN
35	Reticulocytes	RET	Flow cytometry	Sysmex XN
36	Hypersensitive C-reactive protein	hCRP	Immunoturbidimetric	Sysmex XN
37	Neutrophil-to-lymphocyte ratio	NLR	NA	NA
38	Neutrophil / (white blood cell - neutrophil)	dNLR	NA	NA
39	Platelet-to-lymphocyte ratio	PLR	NA	NA
40	Monocyte-to-lymphocyte ratio	MLR	NA	NA

41	Eosinophil-to-lymphocyte ratio	ELR	NA	NA
42	Urea	Urea	UV rate method	Cobas c702
43	Creatinine	Cr	Jaffe's assay	Cobas c702
44	Uric Acid	UA	Colorimetry	Cobas c702
45	Glucose	GLU	Hexokinase method	Cobas c702
46	Kalium	K	Ion selective electrode method	Cobas c702
47	Sodium	Na	Ion selective electrode method	Cobas c702
48	Chlorion	Cl	Ion selective electrode method	Cobas c702
49	Calcium	Ca	NM-BAPTA method	Cobas c702
50	Phosphorus	P	UV endpoint method	Cobas c702
51	Magnesium	Mg	Colorimetry	Cobas c702
52	Serum HCO_3 concentration	HCO3	Enzymic method	Cobas c702
53	Total cholesterol	TC	Cholesterol oxidase method	Cobas c702
54	Triglyceride	TG	Enzymic method	Cobas c702
55	High density lipoprotein cholesterol	HDL-C	Selective inhibition method	Cobas c702
56	Low density lipoprotein cholesterin	LDL-C	Soluble reaction method (SOL)	Cobas c702
57	Total bilirubin	TBIL	Diazo method	Cobas c702
58	Direct (binding) bilirubin	DBIL	Diazo method	Cobas c702
59	Total protein	TP	Biuret method	Cobas c702
60	Albumin	ALB	Bromocresol green method	Cobas c702
61	Globulin	GLB	Calculation method	Cobas c702
62	Albumin to globulin ratio	A/G	Calculation method	Cobas c702
63	Alanine aminotransferase	ALT	Rate assay	Cobas c702
64	γ-glutamyltranspeptidase	GGT	Rate assay	Cobas c702
65	Alkaline phosphatase	ALP	Rate assay	Cobas c702
66	Amylase	AMY	Enzymic method	Cobas c702
67	Lactic dehydrogenase	LDH	Rate assay	Cobas c702
68	Aspartic transaminase	AST	Rate assay	Cobas c702
69	Creatine kinase	CK	Rate assay	Cobas c702
70	Hydroxybutyrate dehydrogenase	HBDH	Rate assay	Cobas c702
71	Creatine kinase-MB subtype	CK-MB	Rate assay	Cobas c702
72	Percentage of glycated albumin	GA%	Enzymic method	Cobas c702
73	Glycated albumin	UGA	Enzymic method	Cobas c702
74	Urine microalbumin	UALB	Immunoturbidimetric	Cobas c702
75	β2-microglobulin	B2-MG	Immunoturbidimetric	Cobas c702
76	Glutathione reductase	GR	Enzymic method	Cobas c702
77	Thymidine kinase 1	TK1	Chemiluminescence	CIS_2
78	Lipoprotein phospholipase A2	LP-PLA2	Chemiluminescence	UPT-3A
79	Thioredoxin	TR	Chemiluminescence	TZD-CL-200E
80	Vascular endothelial growth factor	VEGF	Chemiluminescence	TZD-CL-200E
81	B-natriuretic peptide	BNP	Chemiluminescence	NRM 411
82	B-natriuretic peptide precursor	pro-BNP	Chemiluminescence	Cobas e801

83	Carbohydrate antigen 242	CA242	Chemiluminescence	Cobas e801
84	Carbohydrate antigen 50	CA50	Chemiluminescence	Cobas e801
85	Carcinoembryonic antigen	CEA	Chemiluminescence	Cobas e801
86	Change rate of carcinoembryonic antigen	ΔCEA%	NA	NA
87	Carbohydrate antigen 72-4	CA72-4	Chemiluminescence	Cobas e801
88	Change rate of carbohydrate antigen 72-4	ΔCA72-4%	NA	NA
89	Carbohydrate antigen 125	CA125	Chemiluminescence	Cobas e801
90	Change rate of carbohydrate antigen 125	ΔCA125%	NA	NA
91	Carbohydrate antigen 15-3	CA15-3	Chemiluminescence	Cobas e801
92	Change rate of carbohydrate antigen 15-3	ΔCA15-3%	NA	NA
93	Carbohydrate antigen 19-9	CA19-9	Chemiluminescence	Cobas e801
94	Change rate of carbohydrate antigen 19-9	ΔCA19-9%	NA	NA
95	Alpha fetoprotein	AFP	Chemiluminescence	Cobas e801
96	Change rate of alpha fetoprotein	ΔAFP%	NA	NA
97	Neuron-specific enolase	NSE	Chemiluminescence	Cobas e801
98	Change rate of neuron-specific enolase	ΔNSE%	NA	NA
99	Cytokeratin 19 fragment	CYFRA21-1	Chemiluminescence	Cobas e801
100	Change rate of cytokeratin 19 fragment	ΔCYFRA21-1%	NA	NA
101	Procalcitonin	PCT_2	Chemiluminescence	Cobas e801
102	Troponin T	TnT	Chemiluminescence	Cobas e801
103	Squamous cell carcinoma antigen	SCC	Chemiluminescence	Cobas e801
104	Adrenocorticotrophic hormone	ACTH	Chemiluminescence	Cobas e801
105	Cortisol	Cort	Chemiluminescence	Cobas e801
106	Triiodothyronine	T3	Chemiluminescence	Cobas e801
107	Thyroxine	T4	Chemiluminescence	Cobas e801
108	Free triiodothyronine	FT3	Chemiluminescence	Cobas e801
109	Free thyroxine	FT4	Chemiluminescence	Cobas e801
110	Thyroid stimulating hormone	TSH	Chemiluminescence	Cobas e801
111	Thyroglobulin	TG	Chemiluminescence	Cobas e801
112	Antithyroglobulin antibody	TGAb	Chemiluminescence	Cobas e801
113	Anti-thyroid peroxidase antibody	TPOAb	Chemiluminescence	Cobas e801
114	Parathyroid hormone	PTH	Chemiluminescence	Cobas e801

Table S3. Performances of LR, RF, XGBoost, and LightGBM models.

	LR	RF	XGBoost	LightGBM
Accuracy	74.45%	73.21%	78.19%	79.75%
Precision	73.67%	80.18%	79.28%	81.64%
Recall	65.18%	57.42%	67.96%	70.95%
F1 Score	68.18%	61.03%	71.63%	74.58%
AUC of PD	0.85	0.92	0.94	0.94
AUC of SD	0.78	0.86	0.88	0.89
AUC of PR	0.86	0.92	0.90	0.91
